# Activation of non-classical NMDA receptors by glycine impairs barrier function of brain endothelial cells

**DOI:** 10.1007/s00018-022-04502-z

**Published:** 2022-08-11

**Authors:** Lisa Epping, Christina B. Schroeter, Christopher Nelke, Stefanie Bock, Lukas Gola, Nadine Ritter, Alexander M. Herrmann, Saskia Räuber, Antonia Henes, Beatrice Wasser, Juncal Fernandez-Orth, Winfried Neuhaus, Stefan Bittner, Thomas Budde, Michael Platten, Stjepana Kovac, Guiscard Seebohm, Tobias Ruck, Manuela Cerina, Sven G. Meuth

**Affiliations:** 1 Department of Neurology with Institute for Translational Neurology, University Hospital Münster, Münster, Germany; 2 Department of Neurology, Medical Faculty, Heinrich Heine University Düsseldorf, Düsseldorf, Germany; 3 Department of Cardiovascular Medicine, Institute for Cellular Electrophysiology and Molecular Biology, Institute for Genetics of Heart Diseases (IfGH), University Hospital Münster, Münster, Germany; 4 Competence Unit Molecular Diagnostics, AIT - Austrian Institute of Technology GmbH, Vienna, Austria; 5 Department of Neurology, University Medical Center Mainz, Mainz, Germany; 6 Institute of Physiology I, University of Münster, Münster, Germany; 7 DKTK CCU Neuroimmunology and Brain Tumor Immunology, German Cancer Research Center, Heidelberg, Germany; 8 Department of Neurology, Medical Faculty Mannheim, Heidelberg University, Heidelberg, Germany

**Keywords:** Blood–brain barrier, NMDAR, Glycine, Glutamate, Ca^2+^ signaling, MBMEC

## Abstract

Blood–brain barrier (BBB) integrity is necessary to maintain homeostasis of the central nervous system (CNS). NMDA receptor (NMDAR) function and expression have been implicated in BBB integrity. However, as evidenced in neuroinflammatory conditions, BBB disruption contributes to immune cell infiltration and propagation of inflammatory pathways. Currently, our understanding of the pathophysiological role of NMDAR signaling on endothelial cells remains incomplete. Thus, we investigated NMDAR function on primary mouse brain microvascular endothelial cells (MBMECs). We detected glycine-responsive NMDAR channels, composed of functional GluN1, GluN2A and GluN3A subunits. Importantly, application of glycine alone, but not glutamate, was sufficient to induce NMDAR-mediated currents and an increase in intracellular Ca^2+^ concentrations. Functionally, glycine-mediated NMDAR activation leads to loss of BBB integrity and changes in actin distribution. Treatment of oocytes that express NMDARs composed of different subunits, with GluN1 and GluN3A binding site inhibitors, resulted in abrogation of NMDAR signaling as measured by two-electrode voltage clamp (TEVC). This effect was only detected in the presence of the GluN2A subunits, suggesting the latter as prerequisite for pharmacological modulation of NMDARs on brain endothelial cells. Taken together, our findings argue for a novel role of glycine as NMDAR ligand on endothelial cells shaping BBB integrity.

**Supplementary Information:**

The online version contains supplementary material available at 10.1007/s00018-022-04502-z.

## Introduction

Neurological diseases are often characterized by changes in the balance of neurotransmitter concentrations in the central nervous system (CNS). An excess of glutamate causes excitotoxicity followed by neuronal damage in cerebral ischemia, epilepsy, brain trauma and neurodegenerative disorders [1].

Besides neurons, other non-neuronal cells of the CNS such as endothelial cells (ECs) of the BBB might be exposed to pathological changes in the neurotransmitter milieu. They form the inner lining of blood vessels in the brain and restrict toxins, pathogens and immune cells from entering the CNS [2]. Impairment of BBB function might contribute to the pathophysiological continuum of several neurological disorders [3]. However, the underlying mechanisms inducing barrier dysfunction are still incompletely understood.

The excitotoxic effects induced by excessive glutamate are mainly mediated by activation of *N*-methyl-d-aspartic acid receptors (NMDARs) [4, 5]. NMDARs contribute to excitatory synaptic transmissions in the brain [4]. They can be formed by 7 different subunits (GluN1, GluN2A-D and GluN3A-B) [6]. Two GluN1 subunits are obligatory and assemble into bi- or tri-heteromeric complexes with GluN2 and/or GluN3 subunits. GluN1 and GluN3 subunits contain glycine binding sites, whereas GluN2 subunits are bound and activated by glutamate [6]. For proper channel opening, both binding sites need to be occupied. Most neuronal NMDARs are composed of GluN1 and GluN2A/B subunits. NMDARs formed by these subunits are blocked by Mg^2+^ ions at resting membrane potentials and are primarily permeable for Ca^2+^ ions. In contrast, NMDARs containing GluN3 subunits are insensitive to Mg^2+^ blockade and have reduced Ca^2+−^permeability and response amplitudes [7]. Dysregulation of NMDAR ligands might contribute to the progression of neurological diseases. Interestingly, early studies argued against NMDAR expression on ECs [8, 9], while recent investigations corroborated expression of GluN1, GluN2A and GluN3A by brain endothelium [10, 11].

In this study, we aimed to clarify the role of NMDARs on primary isolated MBMECs. We observed expression of glutamate-insensitive, glycine-responsive NMDARs composed of GluN1, GluN2A and GluN3A subunits. Receptor activation resulted in inward Ca^2+^ current, reduced EC barrier resistance, an impaired migratory capacity, as well as changes in actin distribution.

## Materials and methods

### MBMEC isolation and culture

MBMECs were isolated as previously described from ten 8–12-week-old C57BL/6J mice [12, 13]. Cells were incubated in a humidified incubator with 5% CO_2_ at 37 °C in medium containing 80% Dulbecco’s Modified Eagle Medium (Gibco, 10566016), 20% plasma-derived serum (First Link, 60-00-150), 0.05% basic fibroblast growth factor (Peprotech; PHG0266); 0.1% Heparin (Sigma; H9267) and 4 µg/ml Puromycin (Sigma; P8833). Four days after isolation, puromycin was removed from the medium. Two days later, MBMECs were harvested by trypsinization and seeded for subsequent experiments. If not stated otherwise, all experiments were performed in the media that was used for culturing the cells, containing low concentrations of glycine (0.4 μM). Inflammatory conditions were induced by application of 50 U/ml interferon-gamma (IFN-γ) and tumor necrosis factor alpha (TNF-α) for 24 h.

### Transendothelial electrical resistance (TEER) measurements

Six days after isolation, MBMECs were trypsinized, resuspended and seeded at a density of 2 × 10^4^ cells per transwell insert on an area of 0.47 cm^2^ (pore size 0.4 µm; Corning 141002). Beforehand, transwells were coated with collagen IV (0.4 mg/ml) and fibronectin (0.1 mg/ml) for 3 h at 37 °C. Transendothelial electrical resistance (TEER) measurements were performed using the cellZscope 24-cell module and analyzed with the cellZscope v2.2.2 Software (nanoAnalytics GmbH) as previously described by Kuzmanov et al*.* [14] (for details see corresponding JoVE video). Measurements were taken for four to five days until the MBMEC monolayer reached full confluence (cell layer capacitance at < 1 µF/cm^2^ and TEER at its maximum plateau level). At that point, cells were either treated with vehicle, glutamate (100 µM, 10 mM) and glycine (10 mM) or pre-treated for 1 h with the NMDAR inhibitors 5,7-Dichlorokynurenic acid (5,7 DCKA, 50 µM), 2-Amino-5-phosphonopentanoic acid (AP5, 50 µM), L701.324 (0.1 µM, 1 µM, 10 µM, 50 µM) or the α-amino-3-hydroxy-5-methyl-4-isoxazolepropionic acid receptor (AMPAR) inhibitor perampanel (10 µM) followed by application of glycine (10 mM). All substances were diluted 1:100 in the medium and the TEER was measured for another 24 h.

### Scratch assay

MBMECs were re-seeded at a final density of 1 × 10^4^ cells/well into 96-well flat-bottom (0.32 cm^2^, Corning 3599) and checked daily by light microscopy. When confluent monolayers were formed, MBMEC monolayers were scraped with a p20 pipette tip in a straight line. Thereafter, pictures were acquired using an Axio Scope A1 microscope. The first image of the scratch was acquired immediately after the damage occurred (*t* = 0). Then, plates were placed in a humidified incubator with 5% CO_2_ at 37 °C. Six, eight and ten hours after the first scratch, plates were aligned to the regions photographed at *t* = 0 and new images were acquired. Pictures were quantitatively analyzed using ImageJ v1.45 software. Based on the changes in the area observed over time, a linear function was used to fit the data (*f*(*x*) = y*x + *z*). By using the slope (*y*) as indicator of recovery, migration rates were calculated as described before in μm^2^/*h* (migration rate = *y*/(2*length of the scratch)) [15].

### RNA extraction, cDNA synthesis and quantitative PCR

For mRNA expression analysis on MBMECs, total RNA was extracted using a Quick RNA Micro Prep Kit (Zymo Research). cDNA was synthesized from 300 ng of total RNA using a Maxima First Strand cDNA Synthesis Kit (ThermoFisher Scientific). All experiments were performed according to the manufacturers’ instructions and as described before [16]. For quantitative real-time PCR (qPCR) 4 µl cDNA were used together with Maxima Probe Rox qPCR mix supplemented with mouse GluN1 (Mm00433790_m1), mouse GluN2A (Mm00433802_m1), mouse GluN2B (Mm00433820_m1), mouse GluA1 (Mm00433753_m1), mouse GluA2 (Mm00433753_m1), mouse GluA3 (Mm00497506_m1), mouse GluA4 (Mm00444755_m1) and 18S rRNA (Hs99999901_s1) as endogenous control for TaqMan gene expression assays. For qPCR with QuantiTect Primers 4 µl of cDNA were used together with a SYBR green master mix for GluN2C (QT00127015), GluN2D (QT00154378), GluN3A (QT00290843) and GluN3B (QT00173684) expression assays. 18S rRNA was used as endogenous control. All qPCRs were performed using the StepOnePlus System for 40 cycles (Applied Biosystems). Data were calculated using the change in cycle threshold (ΔCT) compared to the 18sRNA [17].

### Immunocytochemistry

For immunocytochemical stainings, MBMECs were seeded at a final density of 1 × 10^5^ cells/well onto pre-coated coverslips of 12 mm^2^. Cells were fixed with 4% PFA for 10 min at room temperature, washed 3 times for 4 min with PBS. Blocking was performed using a solution with 5% bovine serum albumin, 1% serum and 0.2% Triton-X for 1 h at room temperature. After blocking, cells were washed 3 times for 4 min with PBS and were incubated with the following primary antibodies overnight at 4 °C: rabbit anti-mouse GluN1 (1:100; clone ERP2481(2); abcam ab109182), rabbit anti-GluN2A (1:100; abcam; ab14596), rabbit anti-GluN2C (1:100; clone ERP19094; abcam182277) and rabbit anti-GluN3A (1:100; allomone labs; agc-030). The respective antibodies were diluted in a solution containing PBS and 5% bovine serum albumin. The next day, cells were washed, and incubated for 1 h at room temperature in the dark with the following secondary antibodies: goat anti-rabbit Cy3 (1:500). Finally, cells were covered with 4′,6-Diamidino-2-phenylindole dihydrochloride (DAPI) to counterstain cell nuclei in blue. Images were taken using an AxioScope A1 microscope with an AxioCam camera. Data were analyzed using ImageJ software v1.45 software.

### Calcium imaging

For intracellular calcium ([Ca^2+^]_i_) imaging, cells were cultured on 12 mm^2^ coverslips, coated as described above. Cells were loaded for 30 min with fura-2-AM [5 µM] and 0.005% Pluronic (Sigma Aldrich) in a HEPES-buffered solution (artificial cerebrospinal fluid, containing 125 mM NaCl, 2.5 mM KCl, 1.25 mM NaH_2_PO_4_, 10 mM glucose, 2 mM MgSO_4_, 2 mM CaCl_2_ and 30 mM HEPES, pH 7.35, osmotic concentration of 305 mOsmol/kg). Fluorescence imaging was captured with an epifluorescence inverted microscope equipped with a 40 × oil immersion fluorite objective. For fura-2-AM measurement, we used excitation light from a LED lamp passing through a monochromator at 340 and 380 nm (Cairn Research, Faversham, UK). Fluorescent emission was reflected at 515 nm with a long-pass filter to a charge-coupled device camera (Retiga; QImaging) and digitalized. For imaging analysis, we used MetaFluor Fluorescence Ratio Imaging Software (Molecular Devices, LLC, Canada/US). Ratios were computed between excitation fluorescence at 340 and 380 nm, both with emissions at > 515 nm. Fluorescent data were acquired with a sampling interval of 2 s. [Ca^2+^]_i_ levels were expressed as fura-2-AM ratios. [Ca^2+^] release was measured in response to glycine and glutamate treatment, respectively. Peak [Ca^2+^] responses were calculated by the maximum [Ca^2+^] signal after stimulation subtracted from the baseline [Ca^2+^] signal. Experiments were performed using 8 coverslips from two independent cultures.

### Electrophysiological recordings

Electrophysiological single-cell recordings of MBMEC were performed as previously described [18]. Membrane currents were recorded using a standard patch-clamp setup equipped with an EPC-10 amplifier (HEKA Elektronik) as described before [19]. Borosilicate glass was used to prepare pipettes (GT150T-10; Clark Electromedical Instruments, Pangbourne, UK). The intracellular solution consisted of: 95 mM K-gluconate, 20 mM K3-citrate, 10 mM NaCl, 10 mM HEPES, 1 mM MgCl_2_, 0.5 mM CaCl_2_, 3 mM BAPTA, 2 mM Mg-ATP, and 0.5 mM Na-GTP. The external solution consisted of 125 mM NaCl, 2.5 mM KCl, 1.25 mM NaH_2_PO_4_, 30 mM HEPES, 2 mM CaCl_2_, and 10 mM glucose. Osmolarity was kept at 305 mOsmol/kg constantly. The pH was adjusted with NaOH to a value of 7.35. During the recordings, the pH was maintained bubbling the solutions with carbogen, a combination of 95% O_2_ and 5% CO_2_. The internal solution was kept at a pH of 7.25 with NaOH and an osmolarity of 295 mOsmol/kg. The use of BAPTA, citrate and gluconate for Ca^2+^ buffering, improved stability of measurement and resulted a low free Ca^2+^ concentration. For chelation, we used gluconate and BAPTA for improved stability of measurement. Resistance of glass pipettes and NPC chips (Nanion Technologies) was estimated at 4–7 and 4.5–7 MΩ, respectively. Series resistance was in the range of 4–7 MΩ, and series resistance compensation of > 40% was applied. Glycine and glutamate were added with a pipette at the indicated concentrations directly to the bath in the recording chamber. Perfusion system was kept on hold during administration. The recordings were performed at different time intervals after application of the compounds. The longest interval was 5 min. In this way, we tried to capture fast and long-lasting potential effects without affecting pH and osmolarity of the solution in the recording chamber. We characterized the NMDAR segment by a transient current, caused by the fast kinetics of Ca^2+^ and Na^+^ influx into the cell.

### Molecular biology and oocyte preparation

cRNA of GluN1-1a/pSGEM, GluN2A/pSGEM and GluN3A/pSGEM were generated as previously described [20]. In short, in vitro transcription was performed using mMessage mMaschine T7 kit (Life Technologies, Darmstadt, Germany) and linearized cDNA constructs (PacI for GluN1-1a and GluN3A, NheI for GluN2A). cDNA constructs were kindly provided by Prof. Michael Hollmann (Ruhr University, Bochum). Defolliculated oocytes were purchased from EcoCyte Bioscience (Dortmund, Germany) and injected with 0.8 ng cRNA each for GluN1-1a/GluN2A expression or 10 ng each for GluN1-1a/GluN3A expression using a nanoliter injector 2000 (WPI, Berlin, Germany). Expression of tri-heteromeric NMDA receptors was achieved by injecting 1.6 ng GluN1-1a cRNA and 0.8 ng cRNA each for GluN2A and GluN3A per oocyte. After injection, oocytes were incubated for 4–5 days at 18 °C in Barths solution, containing [mM]: 88 NaCl, 1 KCl, 0.4 CaCl_2_, 0.33 Ca(NO_3_)_2_, 0.6 MgSO_4_, 5 TRIS–HCl, 2.4 NaHCO_3_, supplemented with 80 mg/l theophylline, 63 mg/l benzylpenicillin, 40 mg/l streptomycin, and 100 mg/l gentamycin.

### Compound solutions and two-electrode voltage clamp (TEVC) recordings and analysis

All compounds were provided as 100 mM stock solutions in dimethyl sulfoxide. The compounds were diluted with agonist solution and adjusted to 0.1% dimethyl sulfoxide concentration. Agonist solution was freshly prepared by adding 10 µM glycine and 10 µM l-glutamate to barium ringer solution containing 10 mM HEPES, 90 mM NaCl, 1 mM KCl and 1.5 mM BaCl_2_ (adjusted to pH 7.4 by NaOH). The inhibitory activity was measured via TEVC in *Xenopus laevis* oocytes at room temperature with a holding potential of -70 mV using a Turbo Tec 10CX amplifier (NPI electronic, Tamm, Germany), NI USB 6221 DA/AD Interface (National Instruments, Austin, USA) and GePulse Software (Dr. Michael Pusch, Genova, Italy). Electrodes were backfilled with 3 M KCl and had resistances between 0.5 and 1.5 MΩ. The compounds were tested by applying 50 µM in presence of the agonists, in at least three oocytes.

Data from TEVC measurements were analyzed using Ana (Dr. Michael Pusch, Genova, Italy) and OriginPro 2016 (OriginLab Corporation, Northampton, USA). *p* values were calculated by performing a one-way ANOVA using the Student–Newman–Keuls method (OriginPro). The inhibitory effect of each compound was calculated using the following equation:

$${\text{inhibition}} = 1 - \frac{{I_{c} - I_{h} }}{{I_{a} - I_{h} }}.$$

*I*_*h*_ describes the current without agonists; *I*_*a*_ represents the steady-state current with the agonists present; *I*_*c*_ is defined as the steady-state current in presence of agonist and compound. All dose–response curves were fitted to the following logistic equation:

$$y = \frac{A1 - A2}{{1 + \left( {\frac{x}{{x_{0} }}} \right)^{p} }} + A2.$$

*A*1 describes the minimal inhibition of a compound and was set to 0. *A*2 represents the maximal inhibition of a compound; *p* is the slope of the curve; *x*_0_ is defined as the concentration at half-maximal inhibition and *x* is the tested concentration, respectively.

### Statistical analysis

Each replicate (*n* value) of MBMECs was acquired from a separate culture preparation obtained from 10 mice. Before applying statistical tests, all data was tested for normal distribution via Kolmogorov–Smirnov test and detection of outliers. Comparisons of groups were performed by paired two-tailed student’s *t*-Test, Mann–Whitney or two-tailed Wilcoxon rank-sum test as appropriate. For multiple groups, one-way ANOVA with Turkey’s post hoc or a Kruskal–Wallis or Friedman’s test with Dunn’s post hoc test was applied as appropriate. The alpha level was set at < 0.05 (*p < 0.05, **p < 0.001, ***p < 0.0001) in all cases. GraphPad Prism 9.3 was used to analyze and plot the data. GraphPad Prism 9.3 and Adobe Illustrator were used to illustrate the data.

## Results

### *NMDAR subunits are expressed on MBMECs *in vitro

**Fig. 1:**
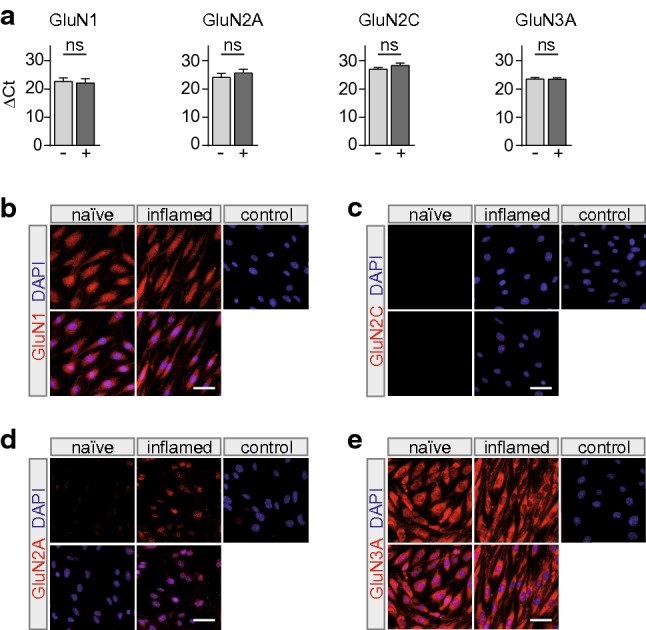
NMDA receptor subunits are expressed on MBMECs in vitro. **a** MBMECs express mRNA coding for the NMDAR subunits GluN1 (*n* = 3), GluN2A (*n* = 6), GluN2C (*n* = 4) and GluN3A (*n* = 5). Expression levels were calculated using the change in cycle threshold (ΔCT) of the target genes compared to the 18sRNA. ΔCT values were determined for naïve MBMECs (–) and MBMECs under inflammation with 50 U/ml IFN-γ, TNF-α for 24 h ( +), respectively. **b–e** Immunocytochemical stainings showing protein expression for GluN1, GluN2A, GluN2C and GluN3A (red) in the cytosol of naïve MBMECs and MBMECs under inflammation with 50 U/ml IFN-γ, TNF-α for 24 h. Nuclei are counterstained with DAPI (blue; representative examples of 3 independent experiments; scale bar 20 µm). The statistical difference between the groups was analyzed with a Wilcoxon matched-pairs signed rank test. The *n* value indicates the number of separate culture preparations, each *n* was obtained from 10 mice. Data are represented as mean ± SEM. *CT* cycle threshold, *DAPI* 4′,6-diamidino-2-phenylindole, *MBMECs* mouse brain microvascular endothelial cells

To gain insight into the role of NMDARs for BBB EC function we first examined receptor subunit expression on primary isolated MBMECs. To understand the influence of inflammatory conditions on NMDAR expression, MBMECs were also analyzed after application of 50 U/ml IFN-γ and TNF-α for 24 h. We found mRNA expression of GluN1, GluN2A, GluN2C and GluN3A subunits on naïve MBMECs and inflamed MBMECs (Fig. 1a). Inflammatory conditions did not induce changes in subunit expression levels of GluN1, GluN2A, GluN2C or GluN3A. No mRNA coding for GluN2B, GluN2D and GluN3B subunits was detected on MBMECs. Next, we wanted to confirm subunit expression on protein level. Therefore, we performed immunocytochemistry staining on naïve MBMECs and MBMECs that were stimulated for 24 h with 50 U/ml IFN-γ and TNF-α. We detected signals for the GluN1, GluN2A and GluN3A subunits (Fig. 1b, d, e), whereas GluN2C was not detectable (Fig. 1c). In conclusion, these data suggest that primary isolated MBMECs express GluN1, GluN2A and GluN3A subunits.

### *Glutamate does not induce NMDAR currents or Ca*^*2*+^*signals in MBMECs nor affect functional properties*

**Fig. 2:**
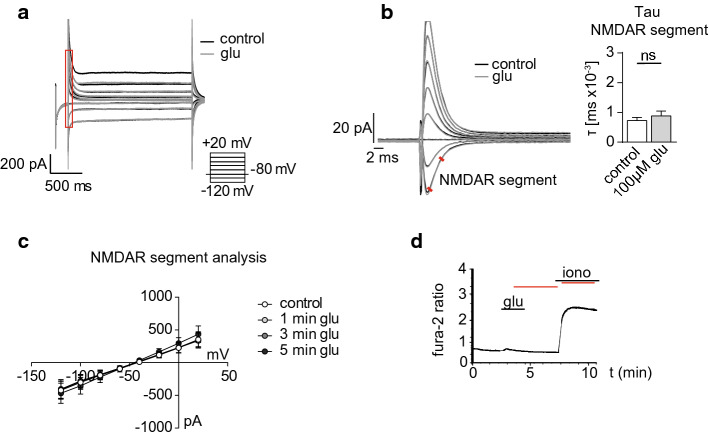
Glutamate treatment does not affect NMDAR-mediated currents and Ca^2+^ signals in MBMECs. **a** Exemplary traces depicting the voltage-clamp protocol used to investigate glutamate-mediated currents in MBMECs. **b** High magnification of the initial segment of the voltage steps as indicated by the red insert in (**a**), after application of 100 µM glutamate (glu; gray) or under control conditions (black) (left). Quantification of the tau of the NMDAR segment as indicated by the red lines (right). **c** Current–voltage relationship graph showing no changes in the current in response to the different voltage steps in the presence of 100 µM glutamate for 1 min (light gray), 3 min (gray) or 5 min (dark gray) and in control conditions (white). **d** Changes of [Ca^2+^]_i_ concentrations in MBMECs were determined using the Ca^2+^-dye fura-2-AM. Ca^2+^ measurements were performed on MBMECs treated with 100 µM glutamate (glu). No differences in [Ca2 +]i were observed upon glutamate application. Ionomycin was used as positive control. A paired student’s *t*-test was performed on **b**. All data are presented as mean ± SEM. *[Ca2 +]i* intracellular calcium, *glu* glutamate, *iono* ionomycin, *MBMECs* mouse brain microvascular endothelial cells

After confirming NMDAR expression on MBMECs in vitro*,* we were interested in the channel functionality of these receptors. Since we detected expression of the conventional NMDAR forming subunits GluN1 and GluN2A, we used the GluN2 site agonist glutamate to induce NMDAR activation and performed voltage-clamp recordings on MBMECs. We were interested if glutamate treatment alone would show the potential to activate NMDA receptors. A combination of Ca^2+^ and Na^+^ currents with a linear *I*/*V* relationship shape, reflecting the literature [21, 22], were recognized as NMDA-mediated currents. The current–voltage relationship showed no changes in glutamatergic currents in response to different voltage steps in the presence of glutamate (100 µM; Fig. 2a–c). Even application of high glutamate concentrations (1.25 mM) did not result into glutamatergic currents (Suppl. Fig. 1a). In line with these findings, administration of AP5, a selective glutamate binding site inhibitor, did also not affect glutamatergic currents recorded in MBMECs (Suppl. Fig. 1b). Additionally, we measured changes in [Ca^2+^]_i_ concentrations using the fluorescent Ca^2+^ indicator dye fura-2-AM. Interestingly, application of glutamate did not induce changes in [Ca^2+^]_i_ (Fig. 2d). Next, we were interested if treatment by glutamate would affect physiological function of MBMEC monolayers. First, we measured the TEER of MBMECs over time in response to glutamate. On *t* = 0 either vehicle, 100 µM or 10 mM glutamate were applied to the cells and the TEER was measured for 24 h (Suppl. Fig. 2a). Glutamate did not affect the TEER of MBMEC monolayers even if applied at high concentrations up to 10 mM (Suppl. Fig. 2b). In accordance with the results obtained from the TEER measurements, investigation of the expression and distribution of the tight junction (TJ) proteins zonula occludens protein 1 (ZO-1) and claudin-5 upon glutamate treatment displayed no differences in comparison to the vehicle-treated control group (Suppl. Fig. 2c). Lastly, we performed scratch assays to examine the migratory capacity of MBMECs in response to glutamate treatment. We scratched MBMEC monolayers, measured the cell free area over time and calculated the rate of repopulation (migration rate) (Suppl. Fig. 2d–f). Glutamate did not affect the migration rate of MBMECs. These data suggest that primary isolated MBMECs express NMDAR that are insensitive to glutamate treatment alone.

### *Glycine induces NMDAR-mediated currents and Ca*^*2*+^*signals in MBMECs*

**Fig. 3:**
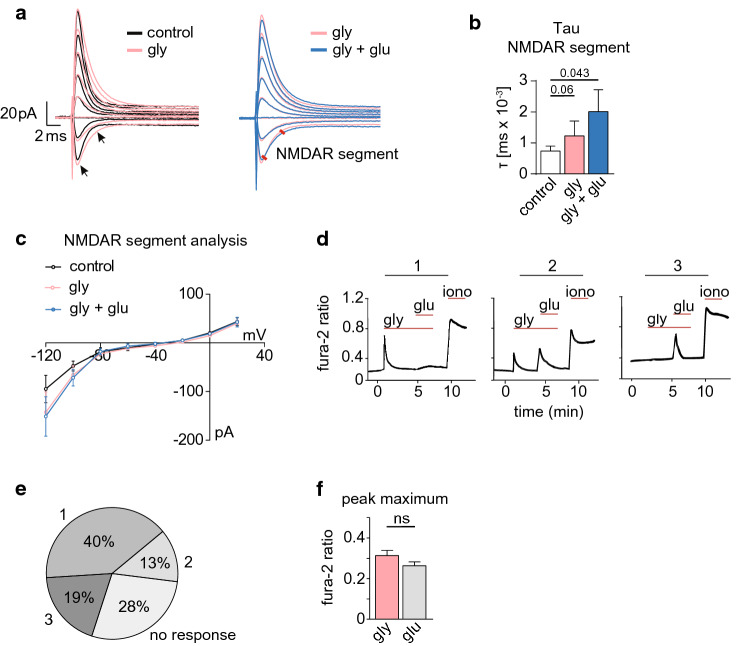
Glycine treatment induces NMDAR-mediated currents and Ca^2+^ signals in MBMECs. **a** High magnification of the initial part of the voltage steps after application of 10 mM glycine (light red) or under control conditions (black; left panel) and after application of 10 mM glycine (light red) or 10 mM glycine + 100 µM glutamate (dark red; right panel). Note the increased amplitude and duration of the red currents as indicated by the black arrows. **b** Quantification of the tau of the NMDAR segment as indicated by the red lines in **a**. **c** Current–voltage relationship graph showing changes in the current in response to the different voltage steps; vehicle (black), glycine (light red), glycine + glutamate (blue). **d–f** Changes of [Ca^2+^]_i_ concentrations in MBMECs were determined using the Ca^2+^-dye fura-2-AM. Ca^2+^ measurements were performed on MBMECs treated with 10 mM glycine followed by application of 5 µM glutamate. Ionomycin [1 µM] was used as positive control. Glycine treatment alone was sufficient to induce and increase in [Ca^2+^]_i_ in 40% of measured MBMECs (1). 13% of MBMECs did not react to glycine but only to subsequent application of glutamate (2). Addition of glutamate evoked a Ca^2+^ response in 19% of MBMECs if it was preceded by glycine (3). Peak maxima of Ca^2+^ signals evoked by glycine and glutamate did not differ (right panel). Friedman’s test with Dunn’s post hoc test was applied in (**b**), *p < 0.05. A paired student’s *t*-test was performed on (**d**). All data are presented as mean ± SEM. *[Ca*^*2+*^*]* intracellular calcium, *iono* ionomycin, *gly* glycine, *glu* glutamate, *MBMECs* mouse brain microvascular endothelial cells, *TEER* transendothelial electrical resistance

Both glutamate and glycine binding contribute to NMDAR signaling [23]. Thus, to investigate whether NMDARs could be activated in the presence of glutamate together with glycine, we measured currents evoked in the presence of glycine (10 mM) followed by the application of glutamate (100 µM) in the recording chamber. We recorded currents at more negative membrane potentials than known for “classical” NMDAR composed of GluN1 and GluN2A subunits (Fig. 3a). Furthermore, the currents were characterized by a greater amplitude and slower kinetics, suggesting the activation of channels with longer opening times (Fig. 3a). Tau-analysis of the segment of the current mediated by NMDAR in order to assess the slope and the opening kinetics supported these findings: Statistics revealed that only the comparison between the control and the glycine + glutamate condition reached significance threshold (*p* = 0.043). Although no statistically meaningful differences were recorded when comparing tau results in control versus glycine-treated MBMECs, a trend towards slower gating kinetics consistent with NMDAR currents was observed (Fig. 3b, c). Interestingly, when glutamate was applied first and then followed by the addition of glycine, no NMDAR-mediated currents were recorded (Suppl. Fig. 3). Last, we investigated the effect of glycine on [Ca^2+^]_i_. Surprisingly, application of glycine alone was enough to induce a calcium signal in MBMECs (Fig. 3d, e). 40% of the cells displayed a [Ca^2+^] response upon glycine treatment but no response on a subsequent glutamate application (group 1; *n* = 52 cells) (Fig. 3d, e). Another 13% of the cells showed a [Ca^2+^] response upon both, glycine and glutamate addition (group 2; *n* = 17 cells). 19% of the cells displayed a peak in [Ca^2+^]_i_ after glutamate application, but only if it was preceded by glycine application (group 3; *n* = 25 cells; Fig. 3d, e). Yet, peak maximum sizes did not differ between glycine- and glutamate-evoked [Ca^2+^]_i_ influx (Fig. 3f). These data support the hypothesis that MBMECs express glycine-responsive NMDARs. Interestingly, we did also detect mRNA expression of the AMPAR subunits GluA1, GluA2, GluA3 and GluA4 on MBMECs (Suppl. Fig. 4a). This finding could explain the variation in Ca^2+^ responses under glycine and glutamate treatment. We cannot exclude the possibility that the increase in [Ca2^+^]i was partly secondary and maybe partly mediated by channel opening of glutamate-sensitive AMPARs. Supporting this hypothesis, treatment of MBMECs with perampanel, an AMPAR inhibitor, lead to a significant increase in TEER of MBMECs under naïve conditions (Suppl. Fig. 4b, c).

### Glycine treatment reduces barrier integrity and migratory capacity of MBMECs

**Fig. 4:**
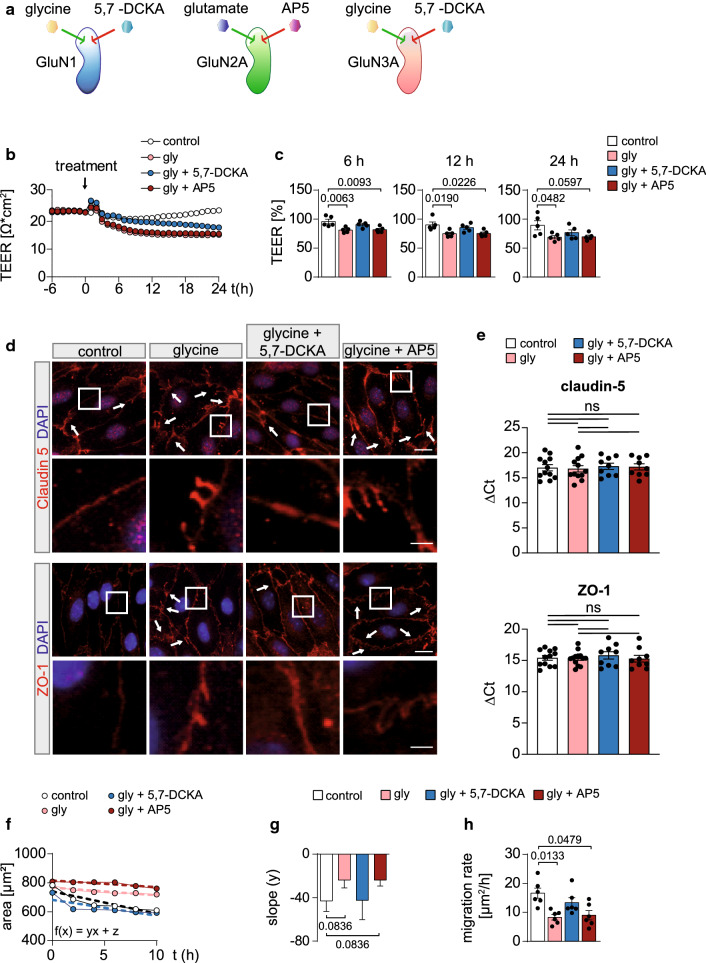
GluN1/GluN3 subunit activation by glycine reduces barrier integrity and migratory capacity of MBMECs. **a** Scheme showing the activation of NMDAR. The GluN2 subunits are activated by glutamate (dark blue) and inhibited by AP5 (red). The GluN1 and GluN3 subunits are activated by glycine (yellow) and inhibited by 5,7-DCKA (green). **b** Representative TEER course of naïve MBMECs. On *t* = − 1 AP5 (50 µM) and 5,7-DCKA were applied on MBMECs. One hour later (*t* = 0), cells were treated with vehicle or glycine (10 mM) and the TEER was measured for 24 h. **c** Scatter plots showing the TEER of MBMECs, normalized to *t* = 0, under vehicle treatment (*n* = 5) or in the presence of 10 mM glycine (*n* = 5), 10 mM glycine + 50 µM AP5 (*n* = 5) or 10 mM glycine + 50 µM 5,7-DCKA (*n* = 5) for 6, 12 and 24 h. **d** Representative immunocytochemistry stainings for claudin-5 (red; upper panel) and ZO-1 (red; lower panel) performed on MBMECs 6 h after application of vehicle or 10 mM glycine, 10 mM glycine + 50 µM 5,7-DCKA (*n* = 5) or 10 mM glycine + 50 µM AP5 (*n* = 5; scale bar: 100 µm). The lower panel is showing the zoom in of the white boxes (scale bar: 25 µm). Nuclei are counter stained with DAPI (blue). **e** mRNA expression levels of claudin-5 and ZO-1 6 h after application of vehicle (*n* = 12) or 10 mM glycine (*n* = 12), 10 mM glycine + 50 µM 5,7-DCKA (*n* = 9) or 10 mM glycine + 50 µM AP5 (*n* = 9). Expression levels were calculated using the change in cycle threshold (ΔCT) of the target genes compared to the 18sRNA. ΔCT values were determined for naïve MBMECs. **f–g** Scratch assays performed on vehicle (*n* = 5), 10 mM glycine (*n* = 5), 10 mM glycine + 50 µM 5,7-DCKA (*n* = 5) or 10 mM glycine + 50 µM AP5 (*n* = 5)-treated MBMECs. A decreased incline of the area size over time is detected under 10 mM glycine and 10 mM glycine + AP5 treatment as demonstrated by slope (*y*) in (**f**). **h** Bar graphs showing a decreased migration rate per hour in 10 mM glycine (*n* = 5) and 10 mM glycine + 50 µM AP5-treated MBMECs (*n* = 5). The statistical difference between the groups was analyzed with a One-way ANOVA with Turkey’s multiple comparison post hoc test. Kruskal–Wallis test was performed on (**e**). The *n* value indicates the number of separate culture preparations, each n was obtained from 10 mice. All data are presented as mean ± SEM. *AP5* 2-Amino-5-phosphonopentanoic acid, *CT* cycle threshold, *5,7-DCKA* 5,7-Dichlorokynurenic acid, *DAPI* 4,6-diamidino-2-phenylindole, *gly* glycine, *glu* glutamate, *MBMEC* mouse brain microvascular endothelial cells, *ns* not significant, *TEER* transendothelial electrical resistance, *ZO-1* zonula occludens protein 1

Next, we wanted to confirm these findings on a functional level by taking advantage of the ability of MBMCS to create tight monolayers resembling BBB functionality. Therefore, we performed TEER experiments under glycine or glycine + glutamate treatment. The transendothelial electrical resistance (TEER) represents the resistance to the ion flux between adjacent endothelial cells and is directly proportional to the barrier integrity: Its decrease hints at a compromised endothelial barrier integrity with increased permeability [14]. We measured the TEER as described by Kuzmanov et al*.* [14] (for details see corresponding JoVE video). First, we performed a titration of glycine and found a significant reduction in TEER upon treatment with 10 mM glycine which did not result in an increased cell death (Suppl. Fig. 5a–c). Next, we were interested if addition of glutamate could potentiate this effect. Therefore, on *t* = 0, 10 mM glycine or 10 mM glycine + 100 µM glutamate were added to the cells and the TEER was measured for 24 h. Interestingly, the additional treatment of glutamate did not potentiate the effect on the TEER previously observed for glycine (Suppl. Fig. 5d, e). Next, we were interested in investigating which subunits were responsible for the reduced TEER as induced by glycine treatment. Therefore, MBMECs were pre-treated for 1 h with the specific glycine binding site inhibitor 5,7-DCKA or the specific glutamate binding site inhibitor AP5 (Fig. 4a). The latter competitively inhibits NMDARs and is commonly used to isolate glutamate-mediated NMDAR currents in heterologous systems of expression, brain slices and in vivo. Subsequently glycine was added and the TEER was measured for 24 h (Fig. 4b, c). Pre-treatment with 5, 7-DCKA but not AP5 could reverse the reduction in TEER as induced by glycine, after 6 h. However, this effect declined over time (Fig. 4b, c). In accordance with these findings, the expression and distribution of ZO-1 and claudin-5 upon glycine treatment were changed when compared to the vehicle-treated control group (Fig. 4d). Glycine induced less continuous TJ borders and the formation of protrusions, detected for both TJ proteins, claudin-5 and ZO-1. Interestingly, pre-treatment with 5,7-DCKA, but not AP5, could rescue the changes in TJ protein distribution induced by glycine (Fig. 4d). However, comparison of mRNA expression levels of claudin-5 and ZO-1 revealed no significant shifts upon glycine treatment (Fig. 4e). To determine whether the migratory capacity of MBMECs was affected by glycine treatment, scratch assays were performed (Fig. 4f–h). Naïve cells were treated with vehicle or glycine (10 mM) or were pre-incubated with 5,7-DCKA (50 µM) or AP5 (50 µM) for 1 h followed by addition of glycine (10 mM). Glycine treatment caused a significant reduction in the migration rate of MBMECs. Again, this effect was rescued by pre-incubation with 5,7-DCKA whereas AP5 treatment did not affect the migration rate (Fig. 4f–h). Surprisingly, application of the GluN1 binding site inhibitor L-701,324 to MBMECs did not reverse the drop in TEER as induced by glycine treatment (Suppl. Fig. 6). These findings, together with the lack of functional effects observed in the presence of AP5, strongly support the hypothesis of NMDAR activation independent of glutamate.

### GluN2A subunit is prerequisite for inhibitory effect of GluN1 binding site inhibitors

**Fig. 5:**
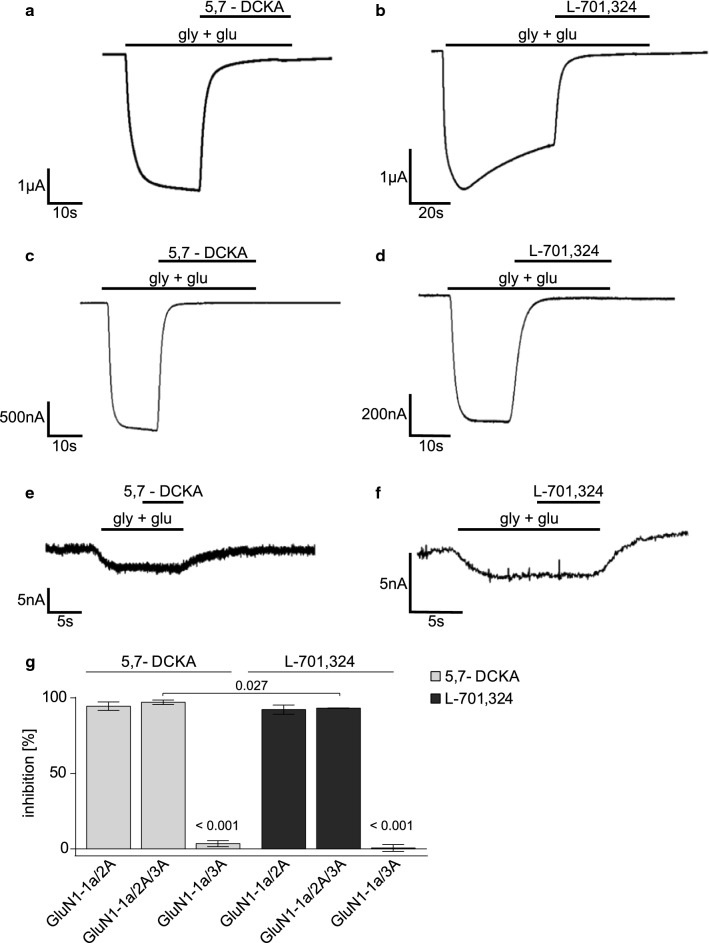
5,7-DCKA and L701,324 inhibit NMDARs composed of GluN1-1a/GluN2A/GluN3A subunits. **a–f** Sample traces of TEVC measurements in oocytes expressing NMDARs composed of GluN1-1a/GluN2A (**a**, **b**), GluN1-1a/GluN2A/GluN3A (**c**, **d**) or GluN1-1a/GluN3A (**e**, **f**) subunits. Application of glycine (10 µM) and glutamate (10 mM) resulted in ion channel activation. 50 µM 5,7-DCKA (**a**, **c**, **e**) or 50 µM L-701,324 (**b**, **d**, **f**) were applied in presence of the two agonists. **g:** Bar graphs showing inhibition of ion currents (%) in TEVC measurements in presence of 10 µM glycine and 10 µM glutamate, caused by application of50 µM 5,7-DCKA (gray) in oocytes expressing GluN1-1a/GluN2A (*n* = 4), GluN1-1a/GluN2A/GluN3A (*n* = 5) or GluN1-1a/GluN3A (*n* = 6). or caused by application of 50 µM L-701,324 (black) in oocytes expressing GluN1-1a/GluN2A (*n* = 3), GluN1-1a/GluN2A/GluN3A (*n* = 3) or GluN1-1a/GluN3A (*n* = 9). One-way ANOVA with the Student–Newman–Keuls method was performed on (**g**). The *n* value indicates the number of separate culture preparations, each *n* was obtained from 10 mice. All data are presented as mean ± SD. *5,7-DCKA* 5,7-Dichlorokynurenic acid, *gly* glycine, *glu* glutamante, *TEVC* two-electrode voltage clamp

To examine the effects of the two GluN1 binding site inhibitors 5,7-DCKA and L-701,324 on different NMDAR subunit compositions in more detail, we performed TEVC measurements on oocytes. NMDAR composed of GluN1-1a/GluN2A (Fig. 5a, b), GluN1-1a/GluN2A/GluN3A (Fig. 5c, d) or GluN1-1a/GluN3A (Fig. 5e, f) were expressed on oocytes and the inhibitory potentials of the two compounds 5,7-DCKA and L701,324 were recorded. Addition of 5,7–DCKA and L701,324 inhibited ion currents mediated by diheteromeric NMDARs composed of GluN1-1a/GluN2A subunits (Fig. 5a, b) as well as tri-heteromeric NMDAR composed of GluN1-1a/GluN2A/GluN3A (Fig. 5c, d). Interestingly, 5,7-DCKA had a significantly higher inhibitory potential on GluN1-1a/GluN2A/GluN3A containing NMDAR than L-701,324 (5,7-DCKA: 97.12 ± 0.68; L-701,324: 92.24 ± 1.78; Fig. 5g). The presence of the GluN2A subunits was prerequisite for the inhibitory effect of both compounds, since diheteromeric GluN1-1a/GluN3A containing NMDARs were neither affected by application of 5,7-DCKA nor L-701,324 (Fig. 5e–g). These results point at the expression of NMDARs composed of GluN1, GluN2A and GluN3 subunits on MBMECs.

### Glycine treatment causes changes in cell morphology and actin distribution

**Fig. 6:**
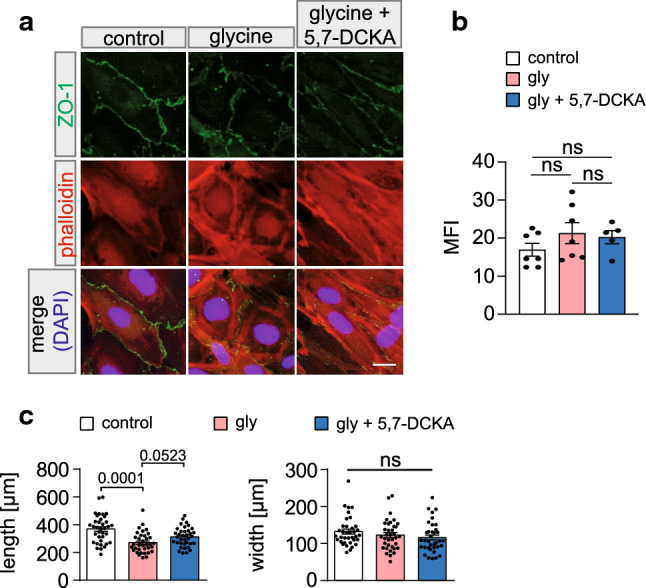
Morphological changes and aberrant actin distribution are induced in MBMECs upon GluN1/GluN3A activation. **a** Representative example of MBMECs stained with ZO-1 (green), phalloidin (red) and DAPI (blue). MBMECs show differences in cell shape and actin distribution upon glycine (10 mM) treatment compared to vehicle and 10 mM glycine + 50 µM 5,7-DCKA-treated MBMECs (1 representative example of 3 independent preparations). **b** Comparison of mean fluorescence intensities (MFIs) of phalloidin staining of MBMECs upon glycine (10 mM) treatment (*n* = 7) compared to vehicle (*n* = 7), and 10 mM glycine + 50 µM 5,7-DCKA (*n* = 5)-treated MBMECs. **c** Analyses of immunocytochemical stainings after 6 h of vehicle, 10 mM glycine and 10 mM glycine + 50 µM 5,7-DCKA treatment. Glycine-treated MBMECs (*n* = 36) are shorter compared to vehicle (*n* = 36) and glycine + 5,7-DCKA (*n* = 36)-treated control cells. A Kruskal–Wallis test was performed on (**b**). A one-way ANOVA with Bonferroni’s post hoc test was performed on (**c**). All data are presented as mean ± SEM. *5,7-DCKA* 5,7-Dichlorokynurenic acid, *DAPI* 4,6-diamidino-2-phenylindole, *gly* glycine, *glu* glutamante, *MFI* mean fluorescence intensity, *TEVC* two-electrode voltage clamp

A loss in barrier integrity and migratory capacity of ECs can be accompanied by changes in actin distribution and polymerization. Under physiological conditions, actin forms a cortical ring under the EC membrane, stabilizing TJ molecules. Reorganization of the actin cytoskeleton can be linked to the activation of NMDAR and changes in [Ca^2+^]_i_ events [24]. Therefore, the distribution of intracellular actin fibers in MBMECs under treatment with vehicle, glycine (10 mM) or glycine + 5,7–DCKA (50 μM) was examined (Fig. 6a). An aberrant distribution of actin in glycine-treated MBMECs was observed, which was reversed by 5,7–DCKA treatment. Here, the cortical actin ring appeared thicker in comparison to control conditions and the cytosolic distribution of actin seemed to be diminished compared to vehicle-treated control MBMECs (Fig. 6a). Comparison of mean fluorescence intensities (MFIs) of phalloidin staining showed no significantly altered polymerization dynamics or formation of stress fibers upon glycine treatment (Fig. 6b). In a last step, we investigated potential morphological changes of MBMECs upon glycine application (Fig. 6c). A reduction in cell body length was observed in MBMECs upon glycine treatment (10 mM) for 6 h. Measuring the length of vehicle-treated MBMECs in comparison to cells treated with 10 mM glycine and 10 mM glycine + 50 μM 5,7–DCKA revealed a significant reduction in cell length under treatment with glycine in comparison to the cell length under treatment with vehicle or glycine + 5,7–DCKA (Fig. 6c). The width of the MBMECs was not affected in any of the experimental conditions (Fig. 6c).

## Discussion

Gathering more insights into NMDAR signaling in ECs might improve our understanding of BBB homeostasis under physiological and pathophysiological conditions. Most knowledge into this topic is gained from experiments performed in cell lines [25, 26]. In this study, we used primary isolated MBMECs to investigate NMDAR expression and function in vitro. In essence, we detected expression of glycine-responsive NMDARs composed of functional GluN1, GluN2A and GluN3A subunits on MBMECs. Interestingly, in contrast to glutamate treatment, glycine treatment alone was sufficient to evoke NMDAR-mediated currents and induce Ca^2+^ signaling in MBMECs. As functional consequence, glycine treatment reduced barrier integrity and migratory capacities of MBMECs. In detail, glycine caused changes in cell morphology and actin distribution linking NMDAR activation and changes of [Ca^2+^]_i_ events to reorganization of the actin cytoskeleton.

Our results show that application of the GluN1 binding site inhibitors 5,7-DCKA and L-701,324 to oocytes, expressing NMDARs composed of different subunits, lead to inhibition of ion currents mediated by NMDAR activation, exclusively in the presence of the GluN2A subunit. These findings are the first to suggest the presence of GluN2A as prerequisite to mediate the inhibitory effects of the two compounds on NMDARs. Additionally, it points at the expression of a novel type of NMDARs on MBMECs, displaying distinct functions from “classical” NMDARs. However, although all three subunits are co-expressed, mixed populations of GluN1/GluN2A, GluN1/GluN3A, and GluN1/GluN2A/GluN3A receptors are present in oocytes and may complicate interpretation of the results [27]. An additional potential complication is that Xenopus laevis oocytes endogenously express XenNR2B which can assemble with GluN1-1a [28]. XenNR2B is weak and causes only small currents in the range of a few nA, which will impact results of robustly expressing GluN1/GluN2A and GluN1/GluN2A/GluN3A receptors only to a small degree. However, as the currents carried by GluN1-1a/3A are in the range of 5–10 nA, it cannot be fully excluded that a significant fraction of the channels contain XenNR2B which would compromise the results. Indeed, 5,7-DCKA was reported to block GluN1/GluN3A currents supporting this notion [29, 30].

Interestingly, Li et al*.* discovered that glycine induces metabotropic activity of GluN2A subunits on rat hippocampal neurons. This leads to activation of ERK1/2 (extracellular signal- regulated kinase 1/2) signaling and in turn, potentiates AMPAR activation, causing an increase in [Ca^**2+**^]_**i**_ [31]. Accordingly, we detected expression of all AMPAR subunits on MBMECs and treatment with perampanel, an AMPAR inhibitor, lead to a significant increase in TEER of MBMECs**.** Therefore, we cannot exclude the possibility, that the increase in [Ca^2+^**]**_i_ upon glycine treatment was, in part, secondary and maybe partly mediated by glutamate-sensitive AMPAR channel opening. The complex interaction between these two receptors could also explain the inconsistent pattern in Ca^**2+**^ influx in MBMECs in response to glycine treatment. Additionally, this could elucidate contradictory data concerning NMDAR signaling on brain ECs described in the literature, especially regarding the role of glutamate in this context, since glutamate also acts on AMPARs. The significance of glutamate signaling in the context of BBB leakage is incompletely understood and intensively discussed [32]. In our study, glutamate appears to affect Ca^**2+**^ signals solely along with glycine, yet no functional consequences on MBMECs are detected if glutamate is present. Thus, more studies are needed to investigate the delicate interplay between the different ionotropic glutamate receptors on brain ECs function.

Integrity of BBB is pivotal to maintain CNS homeostasis under physiological and pathophysiological conditions. However, if dysregulated, BBB breakdown is implicated in propagation of neuroinflammation, such as evidenced for multiple sclerosis, epilepsy, stroke or Alzheimer’s disease [33, 34]. The influence of ion channels on BBB function has been delineated before. We described the endothelial TWIK-related potassium channel-1 as a key mediator for immune-cell trafficking in the CNS [3, 35]. Therefore, it is likely that also other ion channels, such as NMDAR, might affect BBB integrity. Interestingly, NMDA as NMDAR agonist and D-AP5 as NMDAR antagonist, enhance BBB integrity [25], pointing to NMDAR as mediator of BBB integrity. Circulating immune cells, including neutrophils [32], monocytes [36] and T cells [37] were shown to release glutamate in response to inflammatory stimuli, which in turn enabled them to stimulate local NMDAR signaling followed by infiltration of the CNS. However, recent findings indicate that glutamate alone is unable to induce NMDAR-mediated currents on hCMEC/D3 cells and primary mouse brain ECs, while glycine was a potent ligand for NMDAR activation [10]. As previously described, immune cells are able to release glutamate in response to neuroinflammatory conditions. In vivo, high levels of perihematomal glutamate were associated with increased BBB permeability in a rabbit model of intracerebral hemorrhage [38].

In neuroinflammatory and degenerative conditions, microglia and astrocytes are known to be able to release glutamate besides being involved in its reuptake [39–43]. LPS-stressed microglia release glutamate in the brain [44, 45]. Astrocytes releasing glutamate in the vicinity of the BBB may contribute to create an excitotoxic environment [46]. A certain resiliency in responding to fluctuations of glutamate may be an important neuroprotective mechanism. Moreover, Mehra et al*.* showed that NMDAR activation by the agonist NMDA or the co-agonist glycine results in recruitment of the Rho GTPase pathway leading to Rho-associated, coiled-coil containing protein kinase (ROCK)-dependent phosphorylation of myosin light chain (MLC) and increases BBB permeability [10]. Interestingly, it has been reported that in an inflammatory setting, NMDAR ligands, such as glycine and glutamate, as well as tissue-type plasminogen activator (tPA) can be released by infiltrating leukocytes and brain endothelial cells [47–49]. Consequently, the combined effect of glutamate and tPA on brain endothelial NMDARs increases BBB permeability via the Rho GTPase pathway described above [50, 51]. Further, glycine is released from dying cells and has been shown to be elevated in inflammatory conditions like multiple sclerosis [52] or meningitis [53]. Thus, we hypothesize that glycine might promote BBB leakage via NMDAR activation in response to neuroinflammation: Under pathophysiological conditions, inflammation and cell death potentially increase glycine levels, thus weakening BBB integrity and allowing for further immune cell invasion into the CNS, ultimately contributing to a feed-forward loop of self-sustaining neuroinflammation. In respect to the context of pathological conditions, other studies indicated changes in serum and cerebrospinal fluid (CSF) glycine levels: Patients with subarachnoid hemorrhage had significantly higher CSF levels of glycine [54], patients suffering from TBI showed decreased serum concentrations of glycine [55]. Additionally, high glycine concentrations, up to 7.5 mM, have been detected in patients suffering from gliomas, which were associated with a lower patient survival rate and, interestingly, an increase in BBB permeability [56]. Both, glutamate and glycine, can be found in the serum and CSF of patients with different types of multiple sclerosis, Alzheimer’s and Parkinson’s and represent potential biomarkers [52, 57]. Under physiological concentrations, the plasma concentration of glycine is approximately 0.4 mM [58] and the concentration in the CSF is around 0.05 mM [59]. As such, we suspect that the applied concentrations in this study more closely resemble pathological concentrations and interpretation of results should be mindful of this circumstance. In 2019, Skrenkova et al*.* also applied glycine concentrations up to 10 mM before measuring currents of different NMDAR subpopulations, in wild type as well as in mutants [60]. They also performed EC_50_ experiments next to whole-cell patch-clamp recordings from HEK293 cells after treatment with 10 mM glycine [60]. Here, a toxicity of high glycine treatment (10 mM) or desensitization of NMDAR subunits was not observed. Han et al*.* even used 10 mM glycine as standard dose for treatment of wild-type NMDA receptors [61]. Here, functionality of NMDA receptors was proven via treatment with NMDA plus glycine. In the current study, we have performed titration experiments of glycine showing no increase in the number of dead cells in a LDH viability assay after treatment with 10 Mμ, 100 Mμ, 1 mM and 10 mM glycine. Consecutive TEER experiments of naïve MBMECs treated with 10 Mμ, 100 Mμ, 1 mM or 10 mM showed a significant reduction in resistance only after treatment with 10 mM glycine compared to control. The fact that this effect was reversible upon additional treatment with the glycine binding site inhibitor 5,7-DCKA, proves that the observed effects are not artefactual but instead mediated by NMDAR.

In our study, expression of mRNA coding for GluN1, GluN2A, GluN2C and GluN3A was detected while only the expression of GluN1, GluN2A and GluN3A could be confirmed on protein level. Interestingly, there is no consensus concerning the expression of NMDAR subunits on brain endothelial cells. Very few attempts have been done until now to characterize receptors expressed only in ECs of the BBB. Mehra et al*.* [10] stated, by use of immunohistochemistry that endothelial NMDARs contain GluN1, GluN2B and GluN3A subunits. In contrast, there are other reports evidencing expression of functionally relevant GluN2A and GluN2B levels [62–66]. Lu et al*.* [67] detected immunoreactivity for GluN1. Here, other subunits, in particular GluN2C, were recognized by anti-GluN1 immunoprecipitation suggesting structural association. Divergent NMDAR subunit expression profiles may be due to the detection method (immunoblot *vs.* immunoprecipitation *vs.* immunofluorescence staining). Further, conditions of cell cultures employed in the studies at hand might differ, thereby influencing the cellular proteome known to be dynamically adapting to molecular processes, more so than the transcriptome [68]. Besides, different cell lines might introduce biological differences between experimental set-ups. Moreover, one additional condition to take into consideration is the fact that the experiments were performed on primary cell cultures and that, in physiological conditions, these cells and the receptors are normally exposed to different fluctuations of neurotransmitters that, in concert may mediate slightly different mechanisms. Apart from that, residual or contaminating minerals, i.e., from reagents employed in the electrophysiological measurements, could also affect NMDAR recordings, thus, potentially introducing a limitation in our study. The linear NMDAR current analyzed in the present study revealed some similarities to currents observed in cortical astrocytes [21, 22]. A more linear and positive reversal potential has been previously described when glycine was involved in NMDAR activation or depending on the combination of the different subunits [21, 22, 69, 70]. At this point, we cannot exclude a contribution from other channel subpopulations and more complex experiments may be needed in the future to further unravel physiological mechanisms. Importantly, glial NMDARs exhibited a weak Mg^2+^ block at physiological concentrations, revealed a specific pharmacological profile and a tri-heteromeric structure composed of GluN1, GluN2 and GluN3 subunits. Since we found protein expression of GluN1, GluN2A and GluN3A, similar considerations may apply for MBMECs. For di-heteromeric channels composed of GluN1, GluN2A, the Mg^2+^ block was found to have IC_50_ values in the range of tens to hundreds of µM for most of the voltage range investigated here [71]. Therefore, the effect of contamination by ambient Mg^2+^ may be expected to be rather low. In addition, the potentiating effect of ambient glycine may also be considered. These two opposing factors are experimentally difficult if not impossible to control [72] and have to be considered as potential limiting factors in data interpretation.

Our study suggested that NMDAR functionality and actin cytoskeleton dynamics are tightly linked, ultimately regulating the BBB integrity. Consistent with this, recently, Stein et al*.* [73] reported that non-ionotropic NMDAR signaling is essential for bidirectional structural plasticity of dendritic spines. In memory formation in amygdala, NMDAR activation leads to actin regulation via profilin [74]. Vice versa, actin can influence NMDAR activity [75]. Taken together, these data support our hypothesis, that NMDAR signaling on MBMECs affects the actin cytoskeleton and thus MBMEC function.

The scope of our study is limited to investigation of MBMECs in vitro. However, the transfer of experiments to non-CNS ECs might be warranted to deduce specificity. Besides, functional relevance can only be conclusively evaluated in ex vivo or in vivo studies. However, we believe that our findings argue for a role of glycine-sensitive NMDAR on ECs for maintaining BBB integrity. Recently, Macrez et al*.* demonstrated that Glunomab, an antibody directed against the GluN1 subunit of NMDARs, resulted in an ameliorated EAE disease score accompanied by a reduced immune cell infiltration into the CNS [76]. These findings support the hypothesis of NMDAR function for the maintenance of BBB integrity in vivo*.* Additionally, these results also point on an effect of glycine on brain endothelial NMDARs, supporting our hypothesis that glycine-sensitive NMDARs are important for maintaining BBB integrity. Therefore, we can conclude that dysregulated glycine signaling might contribute to BBB leakage and actin cytoskeleton instability under inflammatory conditions. Additionally, we could show that MBMECs seem to express a novel, glycine-responsive type of NMDARs dependent on the presence of GluN2A subunits. However, more detailed research needs to be performed to unravel the exact (patho-)physiological role of these NMDARs for BBB function. Moreover, in vivo studies that investigate glycine-mediated NMDAR signaling on brain ECs are needed and might delineate potential avenues for therapeutic modulation.

### Supplementary Information

Below is the link to the electronic supplementary material.

Supplementary file1 (DOCX 26 KB)

Supplementary file2 (PDF 408 KB)

Supplementary file3 (PDF 1219 KB)

Supplementary file4 (PDF 471 KB)

Supplementary file5 (PDF 498 KB)

Supplementary file6 (PDF 536 KB)

Supplementary file7 (PDF 502 KB)

## Data Availability

The datasets available from the corresponding author on reasonable request.

## Abbreviations

[Ca^2+^]_i_: Intracellular calcium
5,7 DCKA: 5,7-Dichlorokynurenic acid
AMPAR: α-Amino-3-hydroxy-5-methyl-4-isoxazolepropionic acid receptor
AP5: 2-Amino-5-phosphonopentanoic acid
BBB: Blood–brain barrier
CNS: Central nervous system
CSF: Cerebrospinal fluid
CT: Cycle threshold
DAPI: 4′,6-Diamidino-2-phenylindole dihydrochloride
EC: Endothelial cell
ERK1/2: Extracellular signal-regulated kinase 1/2
glu: Glutamate
gly: Glycine
IFN-γ: Interferon-gamma
iono: Ionomycin
MBMEC: Mouse brain microvascular endothelial cell
MFI: Mean fluorescence intensity
MLC: Myosin light chain
NMDAR: NMDA receptor
qPCR: Quantitative real-time PCR
ROCK: Rho-associated, coiled-coil containing protein kinase
TEER: Transendothelial electrical resistance
TEVC: Two-electrode voltage clamp
TJ: Tight junction
TNF-α: Tumor necrosis factor alpha
tPA: Tissue-type plasminogen activator
ZO-1: Zonula occludens protein 1
