# Supplementary material for: Activation of non-classical NMDA receptors by glycine impairs barrier function of brain endothelial cells
Source: Cell Mol Life Sci. 2022 Aug 11;79(9):479. doi: 10.1007/s00018-022-04502-z (PMC9372018; doi:10.1007/s00018-022-04502-z)
Supplement: Supplementary file 1 — Supplementary file1 (DOCX 26 KB) [file 18_2022_4502_MOESM1_ESM.docx]

# SUPPLEMENTARY MATERIAL

**Supplemental Fig. 1 NMDAR-mediated currents on MBMECs are neither affected by high concentrations of glutamate nor GluN2 subunit inhibition**

**a:** Current-voltage relationship graph showing no changes in the current in response to the different voltage steps in the presence of 1.25 mM glutamate for 10min (grey) and in control conditions (black). **b:** Current-voltage relationship graph showing no changes in the current in response to the different voltage steps in the presence of 1.25 mM glutamate for 10min (grey), 25 µM AP5 (red) and in control conditions (black). All data are presented as mean ± SEM.

*glu, glutamante; MBMECs, mouse brain microvascular endothelial cells; NMDAR, NMDA receptor.*

**Supplemental Fig. 2 Glutamate treatment does not affect functional properties of MBMECs**

**a:** Representative TEER course of naïve MBMECs. On t = 0 vehicle or glutamate (glu; 100 µM, 10 mM) were applied and the TEER was measured for 24 hours. **b:** Scatter plots showing the TEER of MBMECs, normalized to t = 0, under vehicle treatment (n = 10) or in the presence of 100 µM glutamate (n = 5) or 10 mM glutamate (n = 5) for 6, 12 and 24 hours. **c:** Representative pictures of immunocytochemistry stainings for Claudin 5 (red; left panel) and ZO-1 (red; right panel) performed on MBMECs 6 hours after application of vehicle or 100 µM glu (scale bar: 100 µm). The lower panel is showing the zoom in of the white box (scale bar: 25 µm). Nuclei are counter stained with DAPI (blue). **d:** Scratch assays performed on vehicle (n = 5) or 100 µM glutamate (n = 5) treated MBMECs. **e:** A similar incline of the area size over time is seen in all groups as demonstrated by the slope (y). **f:** Bar graphs showing no significant differences in the migration rate per hour between vehicle (n = 5) and 100 µM glutamate (n = 5) treated MBMECs (right panel, n=5). The statistical difference between the groups was analysed with a One-way ANOVA with Turkey´s multiple comparison post-hoc test (b) or with a student´s t-test (e, f). The n value indicates the number of separate culture preparations, each n was obtained from 10 mice. All data are presented as mean ± SEM.

*glu, glutamate; MBMECs, mouse brain microvascular endothelial cells; TEER, transendothelial electrical resistance.*

**Supplemental Fig. 3 Preceding glutamate treatment followed by glycine application does not affect NMDAR-mediated currents on MBMECs**

**a:** High magnification of the initial segmentof the voltage steps after application of 100 µM glutamate (grey) or under control conditions (black; left panel) and after application of 100 µM glutamate (grey) or 100 µM glutamate + 10 mM glycine (blue; right panel). **b:** Current-voltage relationship graph showing changes in the current in response to the different voltage steps; vehicle (black), glutamate (grey), glutamate + glycine (blue). All data are presented as mean ± SEM.

*glu, glutamate; gly, glycine; MBMECs, mouse brain microvascular endothelial cells; NMDAR, NMDA receptor.*

**Supplemental Fig. 4 Possible role of AMPA-type glutamate receptors in Ca^2+^ signals in MBMECs**

**a:** Expression of AMPAR subunits GluA1-4 (n = 3 each). Expression levels were calculated using the change in cycle threshold (ΔCT) of the target genes compared to the 18sRNA. ΔCT values were determined for naïve MBMECs (-) and MBMECs under inflammation with 50 U/ml IFN-γ, TNF-α for 24 hours (+), respectively. **b:** Representative example showing the TEER of naïve MBMECs (-) or inflamed MBMECs in the presence of 50 U/ml IFN-γ and TNF-α (+), treated either with methanol vehicle or perampanel (10 µM) for 24 hours. **c:** Bar graphs showing normalized TEER values 6 (n = 5), 12 (n = 5) and 24 (n = 5) hours after perampanel treatment. A significant increase in TEER was observed for naïve MBMECs in the presence of perampanel. All data are presented as mean ± SEM. A paired students T-test was performed on b and c. The n value indicates the number of separate culture preparations, each n was obtained from 10 mice. All data are presented as mean ± SEM.

*CT, cycle threshold; MBMECs, mouse brain microvascular endothelial cells; ns, not significant; TEER, transendothelial electrical resistance*

**Supplemental Fig. 5 Titration of glycine**

**a:** LDH viability assay performed on MBMECs treated with vehicle or 10 mM, 1 mM, 100 µM or 10 µM glycine. No differences in percentage of death cells are seen between the treatments (n = 4). **b:** Representative TEER curve of vehicle-treated MBMECs and MBMECs treated with 10 µM, 100 µM or 1 mM and 10 mM glycine, showing a reduction in TEER only under treatment with 10 mM glycine. **c:** Normalized TEER values 6, 12 and 24 hours after treatment with vehicle or 10 mM, 1 mM, 100 µM and 10 µM glycine. Significant decreases in TEER are seen for all time points under treatment of 10 mM gly (n=5). **d:** Representative TEER curve of vehicle-treated MBMECs and MBMECs treated with 10 mM glycine or 10 mM glycine + 100 µM glutamate, showing a reduction in TEER under treatment with 10 mM glycine. No additional effect of glutamate treatment is detected. **e:** Normalized TEER values 6 and 24 hours after treatment with vehicle, 10 mM glycine or 10 mM glycine + 100 µM glutamate (n = 5). One-way ANOVA with Turkey´s multiple comparison post-hoc test was performed on a, c, and e. *p < 0.05; **p < 0.001. The n value indicates the number of separate culture preparations, each n was obtained from 10 mice. All data are presented as mean ± SEM.

*glu, glutamante; gly, glycine; MBMECs, mouse brain microvascular endothelial cells; NMDAR, NMDA receptor; TEER, transendothelial electrical resistance.*

**Supplemental Fig. 6 Inhibition of the GluN1/GluN3 subunits with L701,324 does not affect TEER of MBMECs**

**a:** Representative TEER course of naïve MBMECs. On t = -1 L701,324 (0.1 µM, 1 µM, 10 µM) was applied on MBMECs. One hour later (t = 0), cells were treated with vehicle or glycine (10 mM) and the TEER was measured for 24 hours (left panel). Scatter plots showing the TEER of MBMECs, normalized to t = 0, under vehicle treatment (n = 3) or in the presence of 10 mM glycine (n = 3), 10 mM glycine + 0.1µM L701,324 (n = 3), 10 mM glycine + 1 µM L701,324 (n = 3) or 10 mM glycine + 10 µM L701,324 (n = 3) for 6, 12 and 24 hours (right panel). **b:** Representative TEER course of naïve MBMECs. On t = -1 50 µM L701,324 were applied on MBMECs. One hour later (t = 0), cells were treated with vehicle or glycine (10 mM) and the TEER was measured for 24 hours (left panel). Scatter plots showing the TEER of MBMECs, normalized to t = 0, under vehicle treatment (n = 4) or in the presence of 10 mM glycine (n = 4) or 10 mM glycine + 50 µM L701,324 (n = 4). Statistical analyses were performed with One-way ANOVA with Turkey´s multiple comparison post-hoc test. The n value indicates the number of separate culture preparations, each n was obtained from 10 mice. All data are presented as mean ± SEM.

*glu, glutamante; gly, glycine; MBMECs, mouse brain microvascular endothelial cells; TEER, transendothelial electrical resistance.*

**Supplemental Table S1.** **Statistics of the inhibition of 50 µM 5,7-DCKA and 50 µM L-701,324 towards GluN1-1a/GluN2A, GluN1-1a/GluN2A/GluN3A and GluN1-1a/GluN3A expressing oocytes**

P values were calculated by performing a One-way ANOVA using the Student–Newman–Keuls method (OriginPro).

| **Compound** | **Subunit** | **5,7-DCKA** | | | **L-701,324** | | |
| --- | --- | --- | --- | --- | --- | --- | --- |
|  |  | **GluN1-1a/2A** | **GluN1-1a/2A/3A** | **GluN1-1a/3A** | **GluN1-1a/2A** | **GluN1-1a/2A/3A** | **GluN1-1a/3A** |
| 5,7-DCKA | GluN1-1a/2A |  | 0.089 | <0.001 | 0.435 | 0.387 | <0.001 |
|  | GluN1-1a/2A/3A |  |  | <0.001 | 0.054 | 0.027 | <0.001 |
|  | GluN1-1a/3A |  |  |  | <0.001 | <0.001 | 0.022 |
| L-701,324 | GuN1-1a/2A |  |  |  |  | 0.614 | <0.001 |
|  | GluN1-1a/2A/3A |  |  |  |  |  | <0.001 |
|  | GluN1-1a/3A |  |  |  |  |  |  |

**Supplemental Table S2.** **Inhibition of ion current in TEVC measurements in GluN1-1a/GluN2A, GluN1-1a/ GluN2A /GluN3A and GluN1-1a/3A expressing oocytes**

Mean inhibition ± SD caused by 50 µM 5,7-DCKA and 50 µM L-701,324 in the presence of 10 µM glycine and 10 µM L-glutamate. The n value indicates the number of separate culture preparations, each n was obtained from 10 mice.

| **Subunit** | **Compound** | **n** | **Inhibition (%)** | **SD (%)** |
| --- | --- | --- | --- | --- |
| GluN1-1a/2A | 5,7-DCKA | 4 | 94.5 | 2.9 |
| GluN1-1a/2A | L-701,324 | 3 | 93.2 | 0.1 |
| GluN1-1a/2A/3A | 5,7-DCKA | 5 | 97.1 | 1.5 |
| GluN1-1a/2A/3A | L-701,324 | 3 | 92.2 | 3.1 |
| GluN1-1a/3A | 5,7-DCKA | 6 | 3.5 | 2.0 |
