# Supplementary figures and images for: Activation of non-classical NMDA receptors by glycine impairs barrier function of brain endothelial cells
Source: Cell Mol Life Sci. 2022 Aug 11;79(9):479. doi: 10.1007/s00018-022-04502-z (PMC9372018; doi:10.1007/s00018-022-04502-z)

**a** NMDAR segment analysis

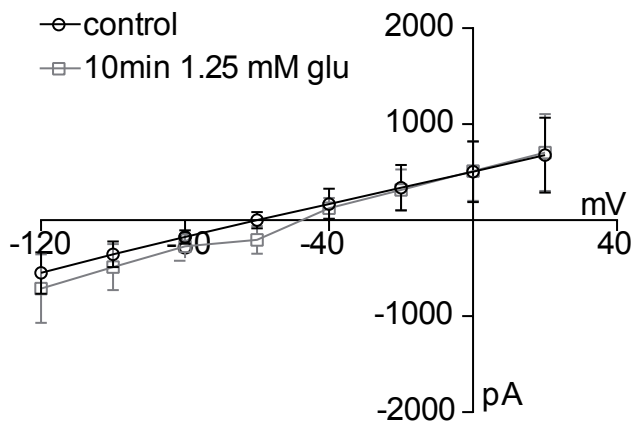

**b** NMDAR segment analysis

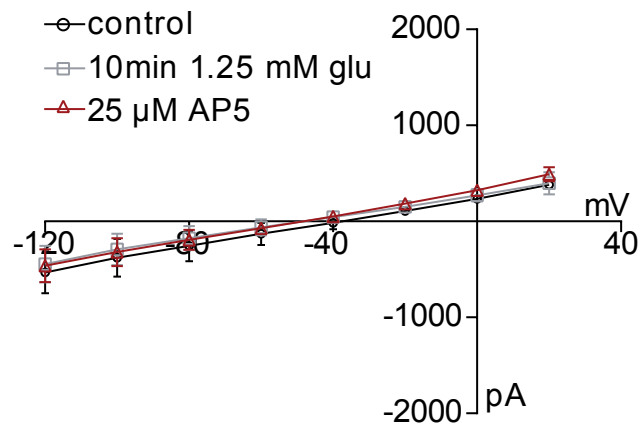

Supplement: Supplementary file 2 — Supplementary file2 (PDF 408 KB) [file 18_2022_4502_MOESM2_ESM.pdf]

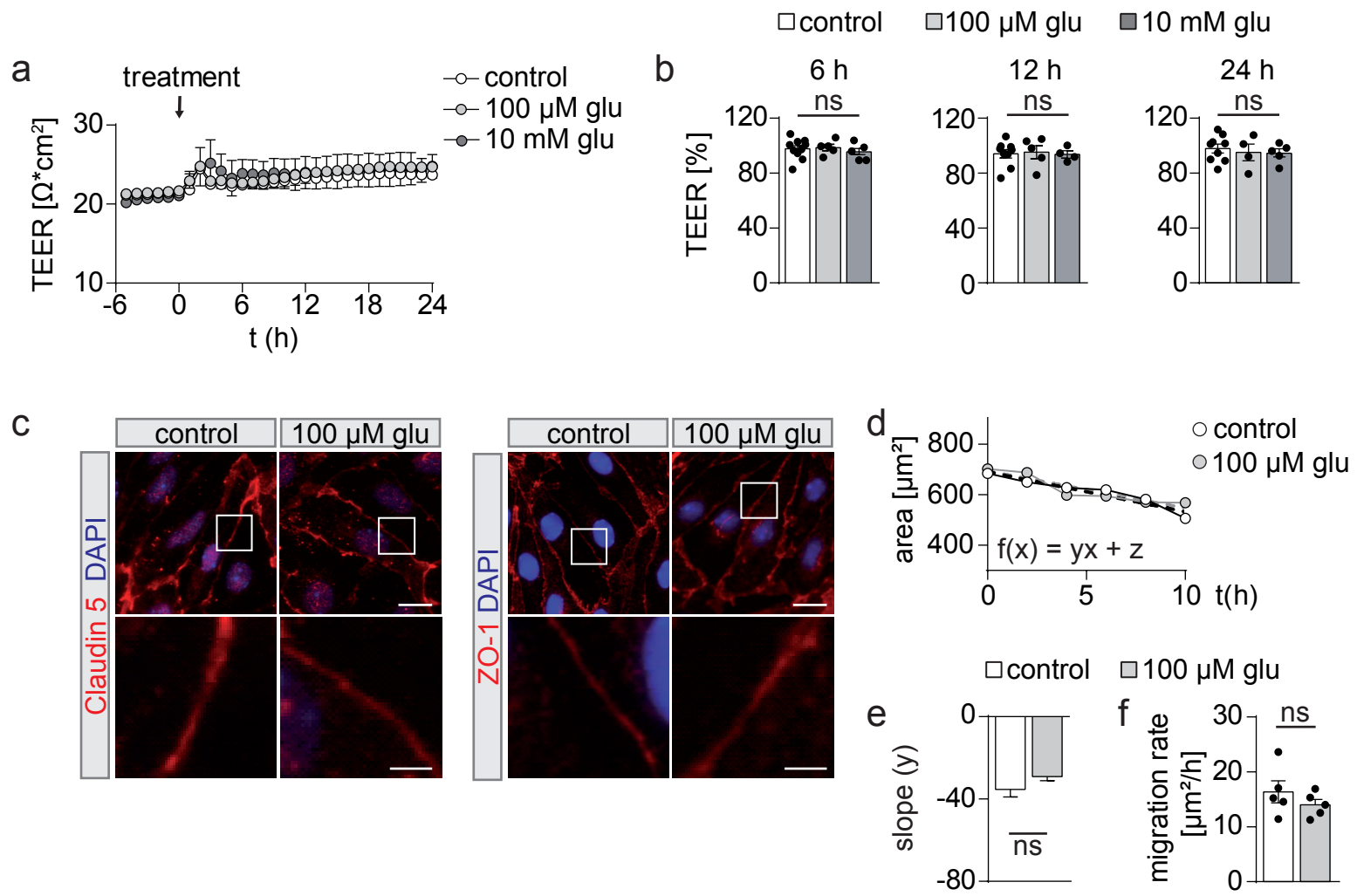

Supplement: Supplementary file 3 — Supplementary file3 (PDF 1219 KB) [file 18_2022_4502_MOESM3_ESM.pdf]

a

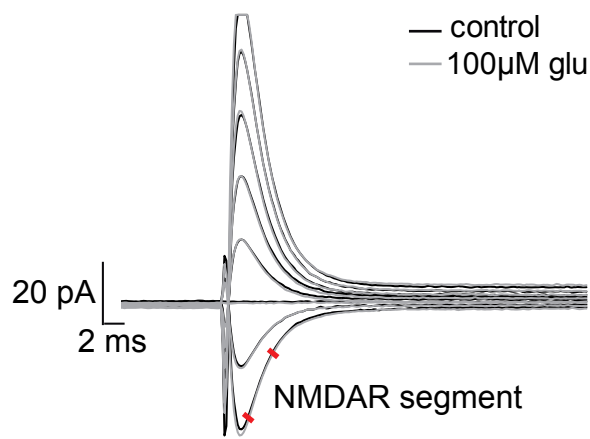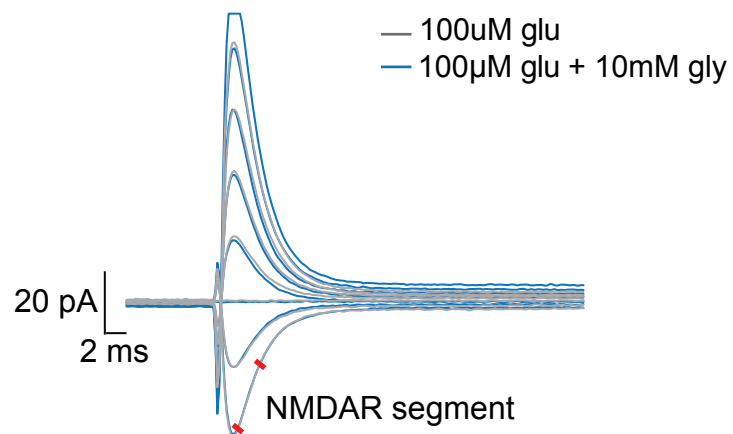

b

NMDAR segment analysis

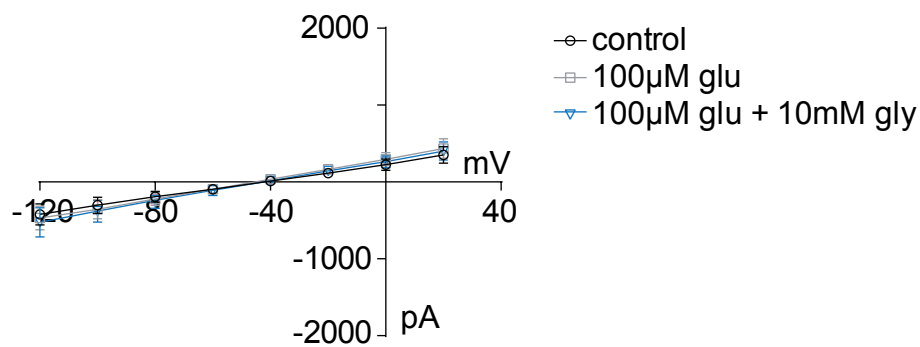

Supplement: Supplementary file 4 — Supplementary file4 (PDF 471 KB) [file 18_2022_4502_MOESM4_ESM.pdf]

**a**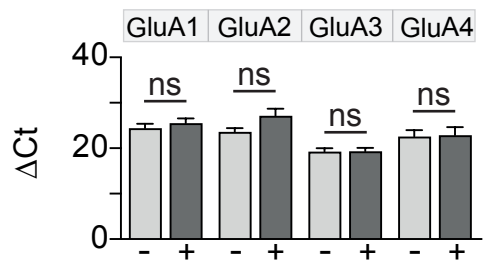**b**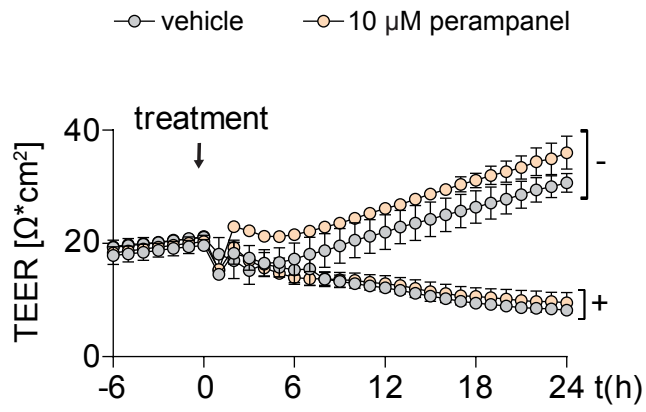**c**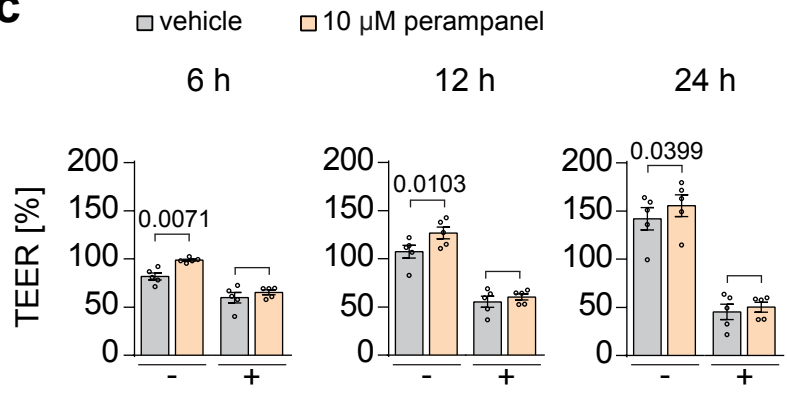

Supplement: Supplementary file 5 — Supplementary file5 (PDF 498 KB) [file 18_2022_4502_MOESM5_ESM.pdf]

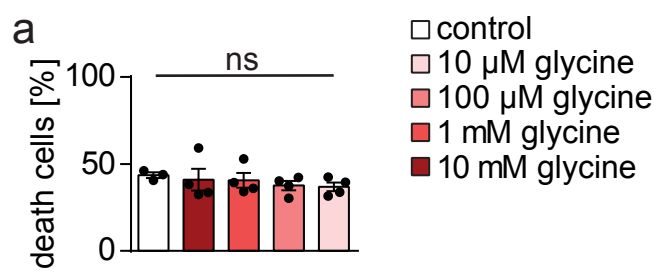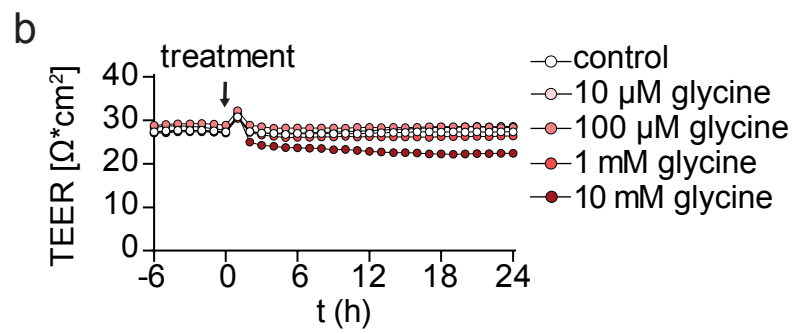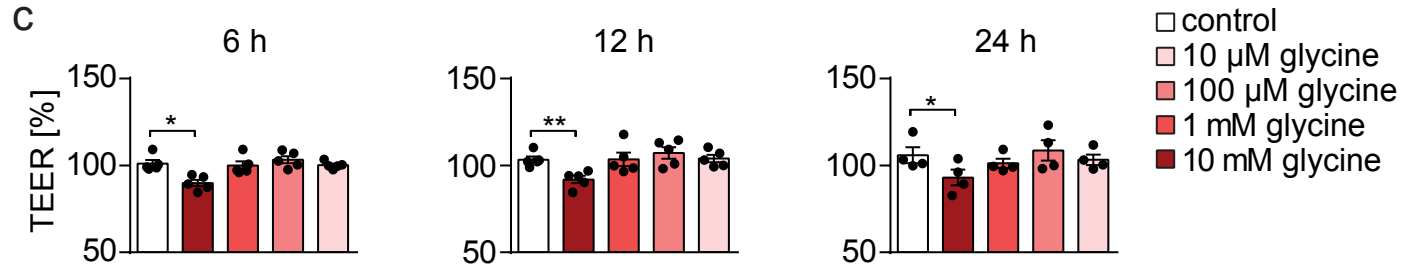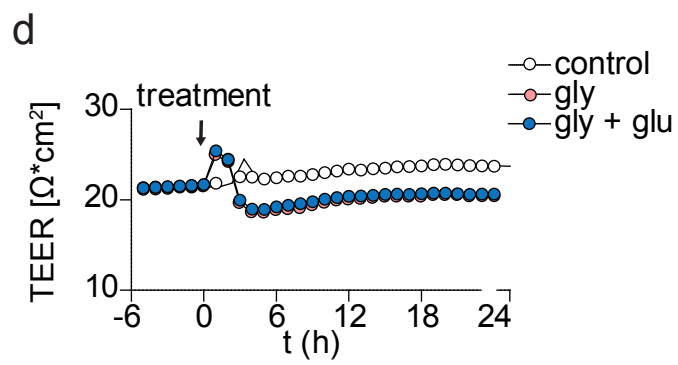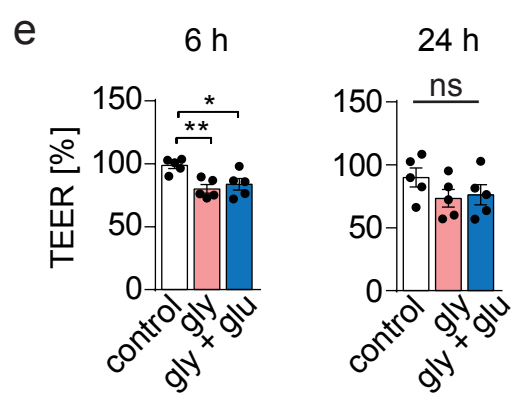

Supplement: Supplementary file 6 — Supplementary file6 (PDF 536 KB) [file 18_2022_4502_MOESM6_ESM.pdf]

a

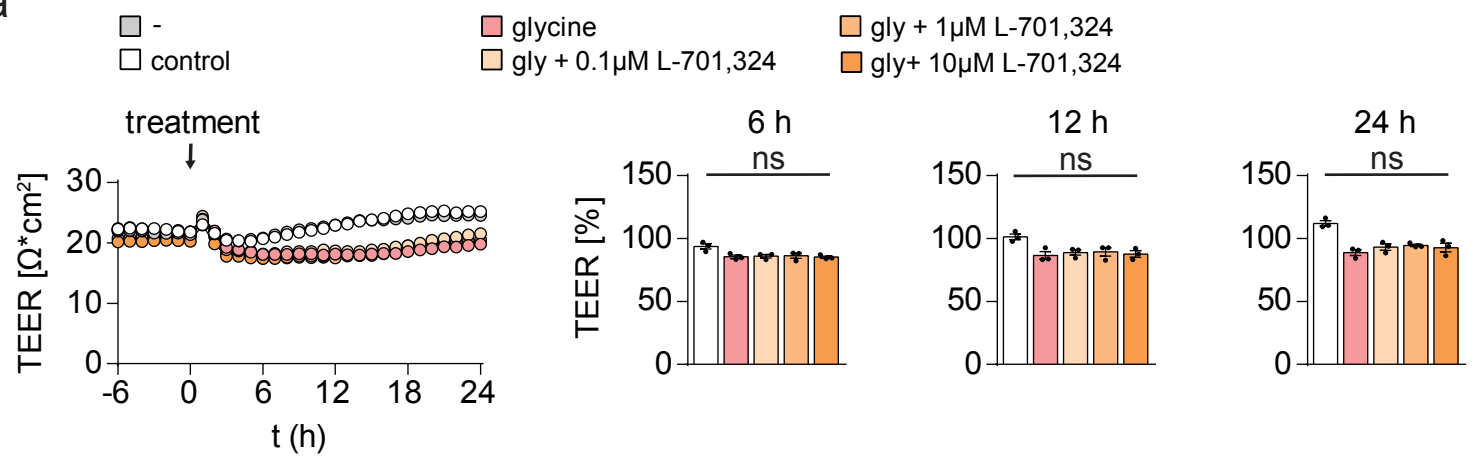

b

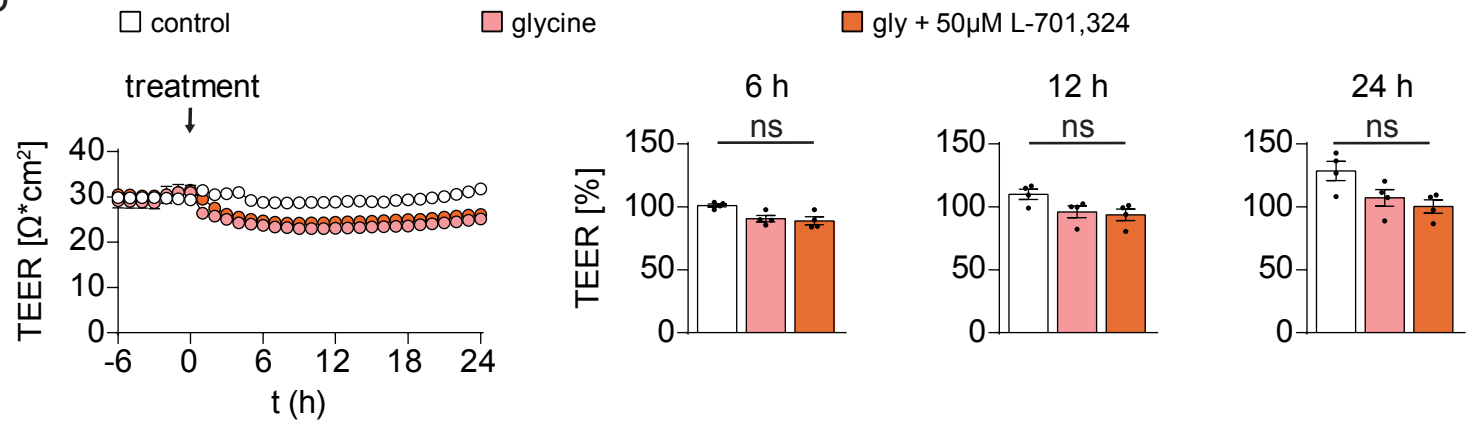

Supplement: Supplementary file 7 — Supplementary file7 (PDF 502 KB) [file 18_2022_4502_MOESM7_ESM.pdf]
